# Split renal function after treatment of small renal masses: comparison between radiofrequency ablation and laparoscopic partial nephrectomy

**DOI:** 10.1177/0284185120956281

**Published:** 2020-09-10

**Authors:** Vanessa Acosta Ruiz, Sarah Båtelsson, Elina Onkamo, Lisa Wernroth, Thomas Nilsson, Maria Lönnemark, Pär Dahlman, Anders Magnusson

**Affiliations:** 1 Department of Surgical Sciences - Radiology, Uppsala University, Uppsala Sweden; 2 Department of Medical Sciences - Molecular Epidemiology, Uppsala University, Uppsala Sweden; 3 Department of Medical Sciences - Nephrology, Uppsala University, Uppsala Sweden

**Keywords:** Kidney, ablation procedures, computed tomography, percutaneous

## Abstract

**Background:**

Radiofrequency ablation (RFA) and laparoscopic partial nephrectomy (LPN) are used to treat small renal masses (SRM; ≤4 cm), although there are conflicting results in the changes in creatinine and estimated glomerular filtration rate (eGFR) after treatment. On contrast-enhanced computed tomography (CE-CT) images, the quantity and quality of renal function can be evaluated by calculating the split renal function (SRF).

**Purpose:**

To compare renal function after RFA or LPN treatment of SRMs through evaluation of the SRF in the affected kidney.

**Material and Methods:**

Single T1a renal tumors successfully treated with RFA (n = 60) or LPN (n = 31) were retrospectively compared. The SRF was calculated on pre-treatment CE-CT images and the first follow-up exam after completed treatment. Serum creatinine and eGFR values were collected simultaneously. To compare renal function outcomes, Student’s t-test and multivariable linear regression models (adjusted to RFA/LPN treatment, pre-treatment SRF/eGFR, BMI, age, tumor characteristics, and Charlson Comorbidity Index) were used.

**Results:**

SRF was reduced in both groups, although reduction was greater in the LPN group (LPN –5.7%) than in the RFA group (RFA –3.5%; *P* = 0.013). After adjusted analysis, the LPN group still had greater SRF reduction (difference 3.2%, 95% confidence interval 1.3–1.5; *P* = 0.001). There was no difference between groups in the change of creatinine/eGFR after treatment.

**Conclusion:**

Both RFA and LPN are nephron-sparing when treating SRMs. However, in this series, reduction of SRF in the affected kidney was smaller after RFA, having a more favorable preservation of renal function than LPN.

## Introduction

Management strategies for localized T1 renal masses include partial nephrectomy (PN), thermal ablation (TA), and active surveillance (1). As survival outcome across treatment strategies is favorable, the preservation of renal function is of paramount importance (1,2). Previous studies report conflicting renal function outcomes, assessed by serum creatinine or glomerular filtration rate (GFR), after PN and TA (3–6). In a meta-analysis (7), a similar change in GFR, incidence of chronic kidney disease, and rates of acute kidney injury for PN and TA are reported. Although creatinine and GFR are used to evaluate renal function for clinical routine purposes, they do not reflect how the treated kidney responds to tumor ablation treatment. When removing a small renal mass (SRM; ≤4 cm), the aim is to preserve nephron mass (and preserve GFR), which might reduce the risk of progression to end-stage renal disease and the need for dialysis (2,8).

Data on whether one modality preserves nephron mass more than the other is lacking. Renal parenchymal volume preservation is reported as more favorable with ablation than with PN (3); however, the quality of nephrons within this volume may not necessarily be homogenous. The split renal function (SRF) can be calculated from contrast-enhanced computed tomography (CE-CT) images, allowing evaluation of the internal renal function ratio between two kidneys (9–14). Hence, both quantity (renal volume) and quality (mean attenuation) of the preserved renal parenchyma are assessed.

The aim of the present study was to compare the renal function preservative properties of radiofrequency ablation (RFA) and laparoscopic partial nephrectomy (LPN) after SRM treatment, through evaluation of the split renal function (SRF) creatinine and eGFR values.

## Material and Methods

### Patient recruitment

The Uppsala regional ethical review board granted approval for the study (Dnr 2012/518). Both RFA and LPN were introduced for treatment of renal tumors at our institution in 2007. Between October 2007 and December 2016, 166 patients with a renal tumor(s) were treated with RFA (in 198 sessions) and 92 patients treated with PN (in 94 sessions). Patient selection for each treatment method and the treatment results and perioperative outcome for the first 97 RFA (91 patients) and 57 LPN (57 patients) consecutive treatments have been previously described (15).

After informed written and verbal consent, patients were assessed retrospectively. Inclusion criteria were: RFA or LPN as primary methods in treating single, T1a, non-hereditary renal tumors originating from the renal parenchyma with a curative intent in patients aged ≤ 75 years. Pre-treatment CE-CT images within one year previously and after treatment were required. The CT images had to meet the technical demands for image processing. Patients needed to have two kidneys in order to calculate the SRF. RFA had to be performed under CT guidance (with the Cool-tip™ RF Ablation System E Series; Medtronic, Boulder, CO, USA) and PN performed with a laparoscopic approach, without conversion to a total nephrectomy. A 100% success rate (i.e. absence of residual tumor or local tumor progression) during the follow-up period (median = 38 months; range = 2.5–99 months) was necessary. Tumors treated by multiple treatment methods were not included. After excluding patients who did not meet these criteria (Supplemental Table), 91 patients were included: 60 patients treated with RFA (treated in 64 sessions) and 31 treated with LPN (in total 31 sessions). Of these, 58 patients were referred (RFA 34, LPN 24) to our institution from hospitals within the region.

### RFA and LPN treatment technique

The RFA and LPN techniques have been previously described (15). Briefly, CT-guided percutaneous RFA applied an ablation margin ≥5 mm; 49 patients were sedated and 11 patient had general anesthesia. RFA treatments were performed by interventional uro-radiologists experienced in CT-guided interventions (AM with 30 years of experience, ML with 20 years, and PD with 10 years).

LPN was performed under general anesthesia using a transperitoneal approach. Tumor resection was followed by suture of the parenchymal defect over a bolster of Surgicel® Original Absorbable Hemostat (Ethicon, Neuchatel, Switzerland). The defect was covered with TISSEEL (Baxter Healthcare Corporation, Westlake Village, CA, USA) for further hemostasis. Two urologists with 25 and 15 years of experience in laparoscopic surgery performed the LPN treatments.

### Data collection and terminology

Patient data (age, gender, body mass index [BMI], updated Charlson Comorbidity Index (16) after excluding the primary renal tumor from the index sum) and tumor characteristics (tumor size, modified RENAL nephrometry score [m-RNS] (17)) were collected retrospectively. Creatinine values before (median = 1 day before treatment) and within one year after completed treatment (median = 310 days for RFA and 225 days for LPN) were collected at the institution or from the referring hospitals. The estimated glomerular filtration rate (eGFR) was calculated using the revised Lund–Malmö formula (18).

Treatment results were reported according to the Society of Interventional Radiology (19) terminology. A renal tumor treatment could include several treatment sessions. Complete tumor treatment was defined as <20 HU of contrast enhancement within the zone where the index tumor was situated (including the surgical/ablation margin).

### CT protocol for pre-treatment planning and patient follow-up

Somatom Definition Flash (Siemens, Forchheim, Germany) was used for the CT examinations. The RFA group underwent CE-CT scans the day before treatment (for treatment planning) and included the unenhanced (UE), corticomedullary phase (CMP), nephrographic phase (NP), and excretory phase (EP) (with contrast medium Iomeron 400 mg I/mL iomeprol, 1  mL/kg, maximum 80 mL, Bracco Imaging SpA, Milano, Italy). The LPN group had CE-CT scans (in the same phases) before treatment. Follow-up imaging included new CE-CT scans (at the institution or the referring hospitals) in the same phases at three months (RFA group only), six months, 12 months, and then yearly after treatment for a minimum of five years.

### Image analysis and split renal function measurement

**Fig. 1.**
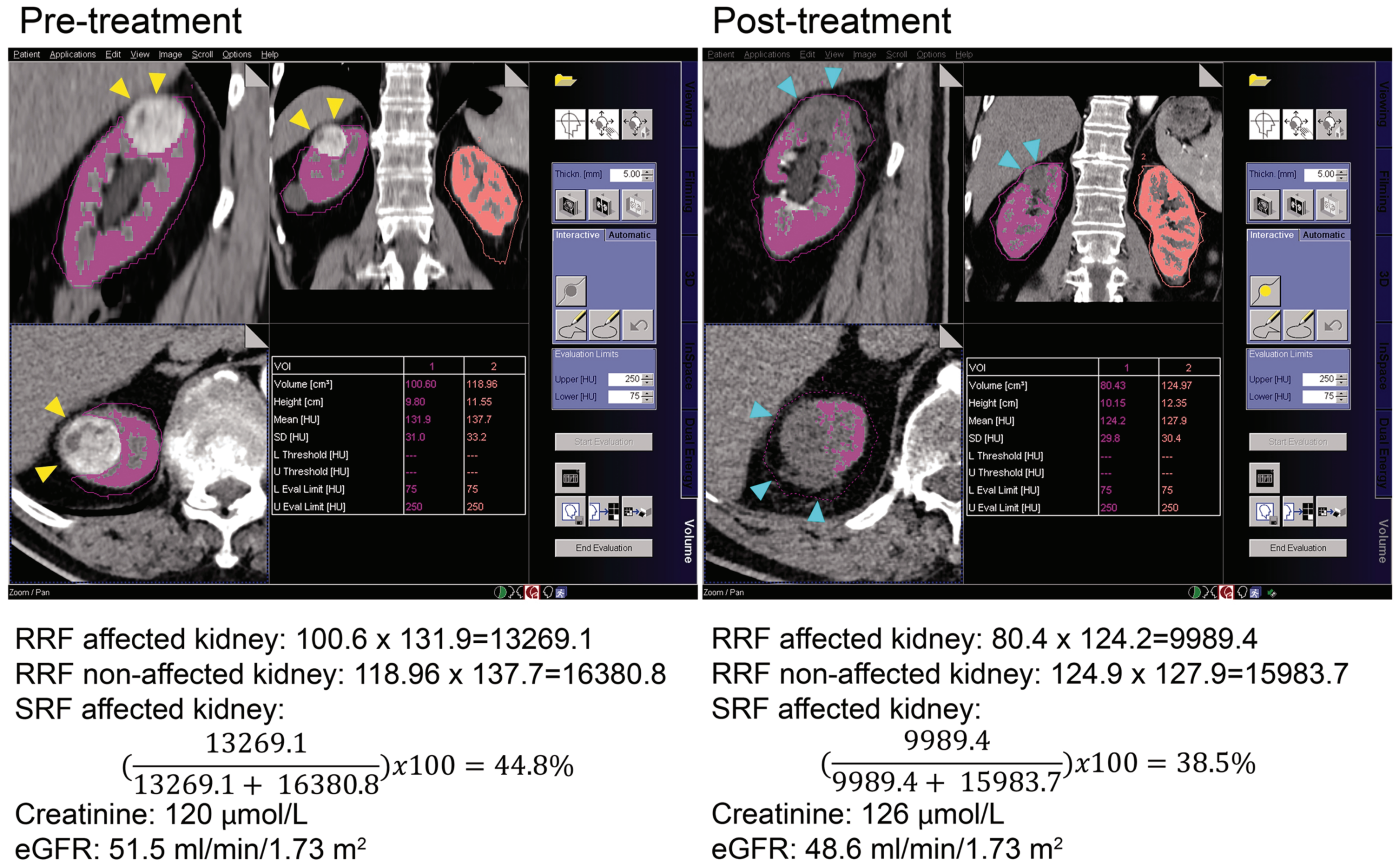
Example of SRF calculation, creatinine, and eGFR values before and after treatment. Yellow arrows point at the tumor before treatment. Blue arrows point at the ablated renal defect after RFA treatment. eGFR, estimated glomerular filtration rate; RFA, radiofrequency ablation; SRF, split renal function.

Measurements of SRF obtained from CE-CT images give comparable results to those acquired from renal scintigraphy (9–14). The SRF calculation was based on the method used by Nilsson et al. (11) and Björkman et al. (9,13). The MultiModality workstation (Siemens, Forchheim, Germany) and the program “Volume” were used for retrospective image analyses of CMP or NP images with a slice thickness of 5 mm (9). On the pre-treatment CE-CT image, regions of interest (ROI) were placed manually in axial sections throughout the length of each kidney. The inclusion limits of the ROI tool were set at 75–250 HU to include only contrast-enhancing renal parenchyma. Structures that did not contribute to the filtration process (i.e. blood vessels, collecting system, ureter and renal tumors) were excluded from the ROI. From each ROI, the volume (cm^3^) and mean attenuation (HU) of each kidney was automatically computed and registered (Fig. 1).

The SRF of the affected kidney was obtained by the formula:

$$\mathrm{Relative}\;\mathrm{renal}\;\mathrm{function}\;\left(\mathrm{RRF}\right)=\mathrm{renal}\;\mathrm{volume}\;\left({cm}^{3}\right)\times \mathrm{mean}\;\mathrm{attenuation}(HU)$$

SRF affected kidney

$$\;=\frac{\mathrm{RRF}\;\mathrm{affected}\;\mathrm{kidney}}{\mathrm{RRF}\;\mathrm{affected}\;\mathrm{kidney}+\;\mathrm{RRF}\;\mathrm{non}\;\mathrm{affected}\;\mathrm{kidney}}$$

Post-treatment SRF analysis was performed on the first follow-up image after completed treatment, carefully excluding any operative material (e.g. surgical clips, hemostatic agents) placed after LPN. Image analyses and SRF measurements were performed by a resident in radiology (VA) and a medical student (SB) blinded from each other. As there was a high inter-rater agreement in SRF measurements between the two observers (intraclass correlation coefficient [ICC] average = 0.997; 95% confidence interval [CI] = 0.997–0.998), the mean value of the two observers’ measurements was used for further SRF calculations. The primary endpoint was the SRF change in the affected kidney (from pre- to post-treatment) in percentage points. Secondary endpoints were changes in creatinine and eGFR values.

### Statistical analyses

For comparison of pre-treatment characteristics between the LPN and RFA groups, Fisher’s exact test was used for categorical variables and Student’s t-test was used for continuous variables. To assess the inter-rater reliability for SRF ICC, estimates with 95% CIs were calculated with the SPSS statistical package version 23 (SPSS Inc., Chicago, IL, USA) and based on a mean-rating (k = 2), absolute-agreement, two-way mixed-effects model.

Treatment effects were compared with Student’s t-test and multivariable linear regression models, and both crude and adjusted mean differences with 95% CI are reported. Three separate regression models were fitted with the change (post-value – pre-value) in SRF, eGFR, and creatinine as the response variables. Treatment (LPN/RFA), the pre-value of the response variables, and confounders (pre-treatment eGFR, BMI, age, tumor size, tumor nearness [distance to the collecting system or sinus] and Charlson Comorbidity Index) were included in the models as explanatory variables. Confounders were selected for adjustment based on prior knowledge regarding their effect on renal function and choice of treatment. All analyses except the calculation of ICC were performed using SAS software, version 9.4 (SAS Institute, Inc., Cary, NC, USA), except the calculation of ICC. All statistical tests were two-tailed with a significance level of 0.05.

## Results

In the RFA group, 56 patients were treated in a single session and four patients required two ablation sessions to achieve complete tumor treatment. All the patients in the LPN group were treated in a single session. The patients in the RFA group were older, but there was no significant difference in gender distribution, mean BMI, CCI, or tumor characteristics between the two groups (Table 1). Tumor histopathology for each treatment group is presented in Table 1.

**Table 1.** Patient and tumor characteristics distributed according to treatment.

	RFA (n = 60)	LPN (n = 31)	*P* value*
Patient characteristics			
Male	37 (61.7)	21 (67.7)	0.65
Age (years)	65.0 ± 9.8 (28–75)	57.8 ± 12.4 (32–75)	0.003
BMI (kg/m^2^)	27.8 ± 5.3 (16.8–45.2)	28.6 ± 4.4 (17.4–38.8)	0.50
CCI			0.18
0	30 (50.0)	23 (74.2)	–
1	8 (13.3)	1 (3.2)	–
2	19 (31.7)	7 (22.6)	–
3	2 (3.3)	0 (0.0)	–
4	1 (1.7)	0 (0.0)	–
Tumor characteristics			
Tumor diameter (mm)	27.1 ± 6.6	28.6 ± 6.9	0.32
Tumor nearness (to the collecting system or sinus, mm)	5.8 ± 6.0	8.8 ± 8.1	0.055
m-RNS (points)	6.8 ± 1.8	6.5 ± 1.8	0.40
Tumor complexity (m-RNS points)			
Low (4–6 points)	28 (47)	15 (48.5)	–
Medium (7–9 points)	26 (43)	14 (45)	–
High (10–12 points)	6 (10)	2 (6.5)	–
Histopathology			
Oncocytoma	6 (10)	5 (16.1)	
Clear cell RCC	27 (45)	20 (64.5)	
Papillary RCC	7 (11.6)	4 (13)	
Chromophobe RCC	8 (13.4)	1 (3.2)	
Other cancer	4 (6.7)	1 (3.2)	
Non-diagnostic biopsies	8 (13.3)	–	

Values are given as n (%) or mean ± SD (range).

*P value from Student’s t-test for continuous variables, Fisher’s exact test for categorical variables.

BMI, body mass index; CCI, Charlson Comorbidity Index; LPN, laparoscopic partial nephrectomy; m-RNS, modified RENAL nephrometry score; RCC, renal cell carcinoma; RFA, radiofrequency ablation; SRF, split renal function.

**Fig. 2.**
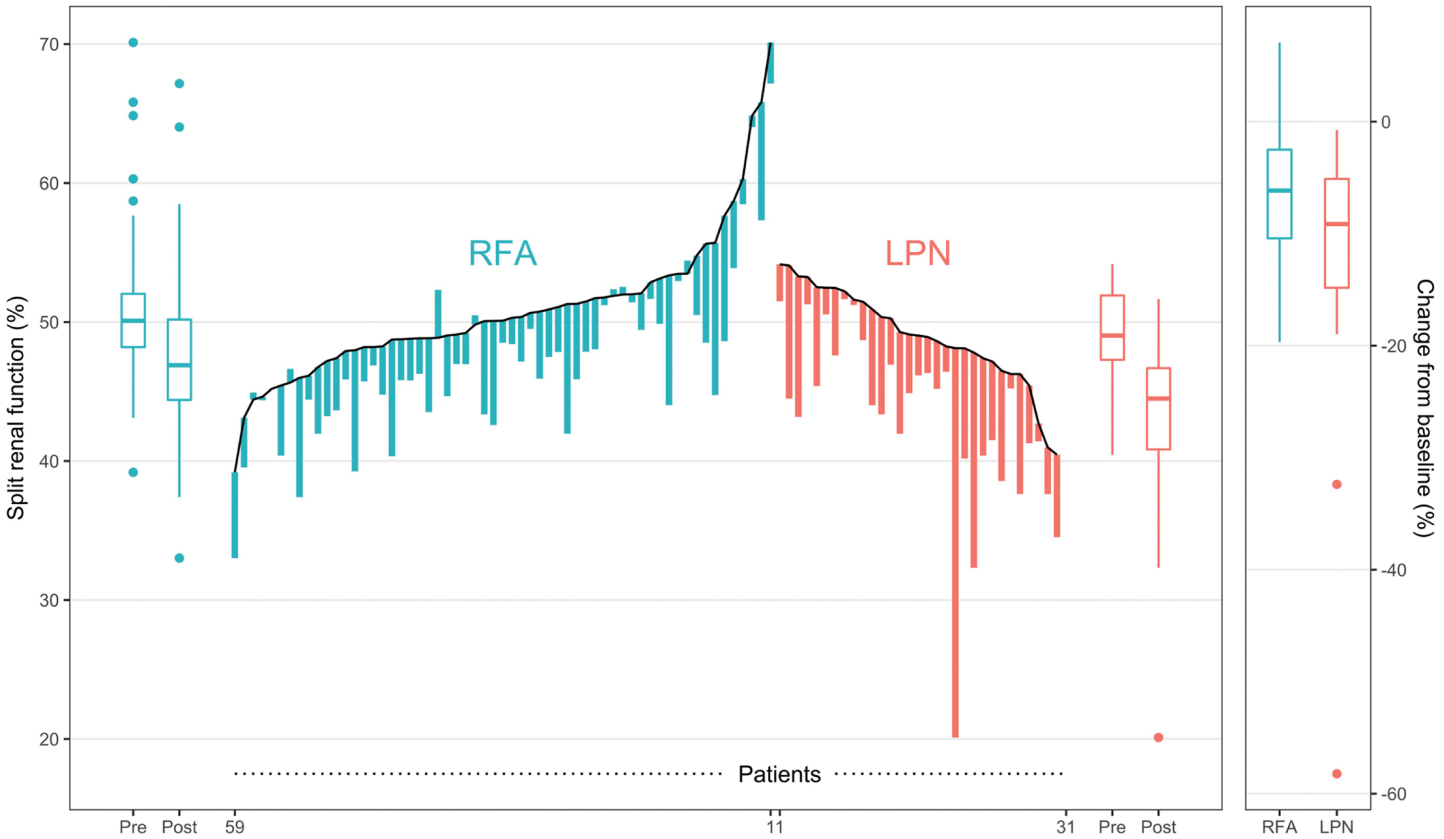
Hybrid parallel line plot showing RFA and LPN patients’ pre- and post-treatment SRF values of the affected kidney. RFA, n = 60; LPN, n = 31. LPN, laparoscopic partial nephrectomy; RFA, radiofrequency ablation; SRF, split renal function.

There was no difference in pre-treatment SRF between the two groups (Table 2). SRF was reduced in both groups after treatment (Fig. 2); however, reduction of SRF was greater in the LPN group (–5.7%) than in the RFA group (–3.5%: difference = 2.2 percentage points, *P* = 0.013; Table 2). After adjustment analysis, the LPN group still had a greater reduction of SRF than the RFA group (Table 2). In other words, the SRF was 3.2% lower in the LPN group than in the RFA group (*P* = 0.001).

**Table 2.** Pre-treatment, post-treatment and change in renal function by treatment method.

	RFA (n = 60)	LPN (n = 31)	Difference RFA – LPN	*P* value*	Adjusted difference (RFA – LPN)	*P* value^†^
SRF affected kidney (%)						
Pre-treatment	50.8 (49.4–52.1)	49.0 (47.7–50.3)	1.7. (-0.4–3.8)	0.10		
Post-treatment	47.3 (45.8–48.8)	43.3 (40.9–45.7)	4.0 (1.3–6.7)	0.004		
Change post–pre	−3.5 (–4.3– –2.7)	−5.7 (–7.7– –3.8)	2.2 (0.5–4.0)	0.013	3.2 (1.3–5.1)	0.001
*P* value	<0.001	<0.001	–	–		
Serum creatinine (μmol/L)						
Pre-treatment	80.5 (75.2–85.8)	78.8 (72.5–85.1)	1.7 (–6.8–10.2)	0.69		
Post-treatment	84.7 (79.5–89.9)	82.8 (74.7–90.8)	1.9 (–7.3–11.2)	0.68		
Change post–pre	+4.2 (1.3–7.1)	+4.6 (0.9–8.2)	−0.4 (–5.3–4.5)	0.87	−0.25 (−5.08–4.58)	0.92
*P* value	0.006	0.015				
eGFR (mL/min/1.73 m^2^)						
Pre-treatment	71.1 (67.6–74.7)	78.1 (72.1–84.1)	−6.9 (–13.4– –0.5)	0.034		
Post-treatment	68.3 (64.6–72.0)	75.3 (68.6–81.9)	−6.9 (–13.9–0.03)	0.051		
Change post–pre	−2.8 (–4.8– -0.8)	−3.3 (–6.1– –0.5)	0.5 (–2.9–3.9)	0.76	−0.01 (–3.3–3.3)	0.99
*P* value	0.006	0.022				

Values are given as mean (95% CI).

*P values from Student’s t-test.

^†^Adjusted mean difference estimated by linear regression model with change (post – pre-value) as the response variables. Treatment (LPN/RFA), the pre-value of the response variable, BMI, age, tumor nearness and Charlson Comorbidity Index were included in the models as explanatory variables.

CI, confidence interval; eGFR, estimated glomerular filtration rate; LPN, laparoscopic partial nephrectomy; RFA, radiofrequency ablation; SRF, split renal function.

**Fig. 3.**
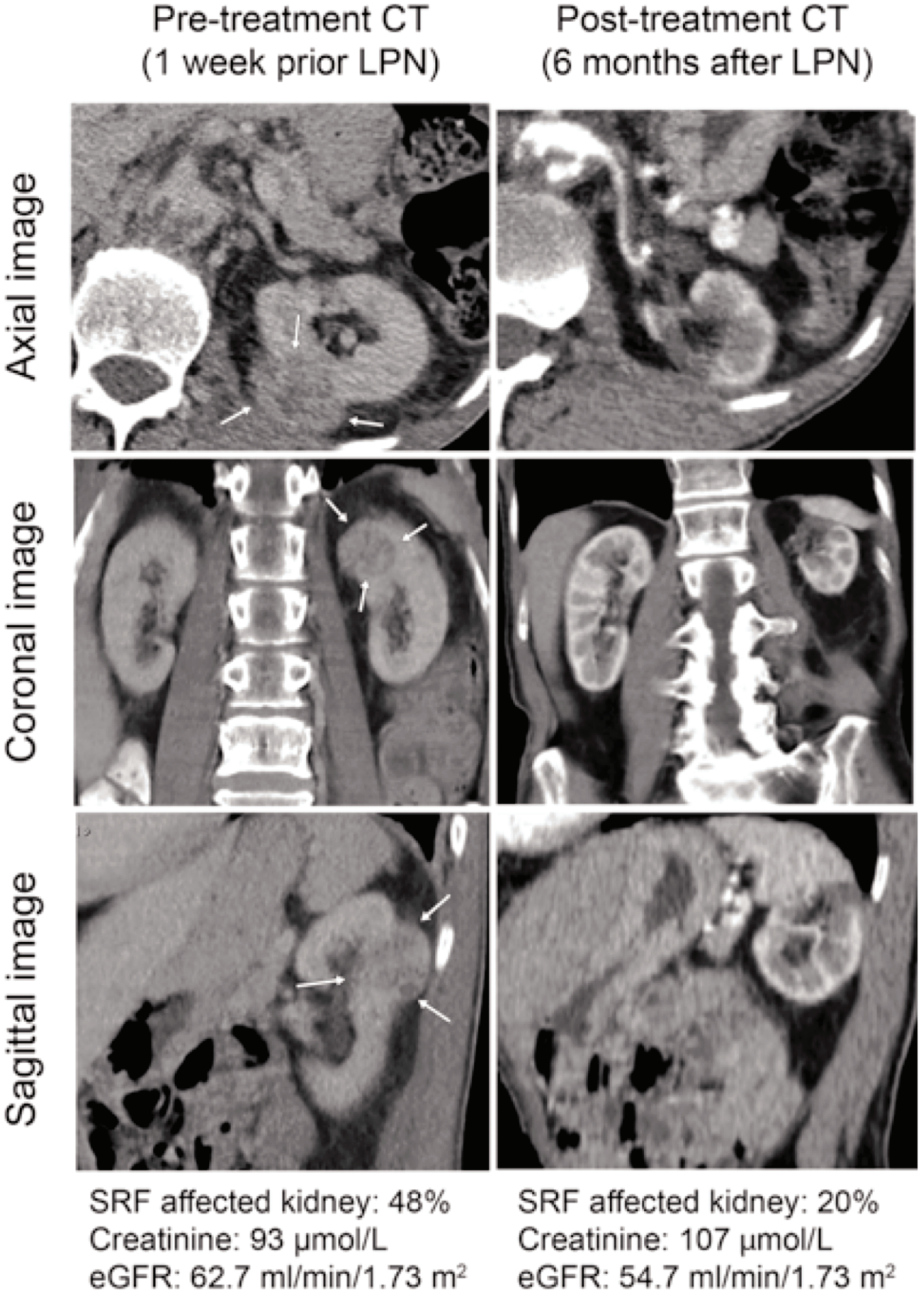
Example of one of the outliers in the LPN treatment group demonstrating unexpected pronounced reduction in split renal function. CT images and renal function values before and after treatment of a 3.6 cm renal tumor in the dorsal aspect of the left kidney (m-RNS = 8p). Warm ischemia time = 30 min. Arrows point to tumor before treatment. CT, computed tomography; LPN, laparoscopic partial nephrectomy; mRNS, modified RENAL nephrometry score.

There were two outliers in the LPN group with an unexpectedly pronounced reduction in SRF (Fig. 2). Due to adhesions from previous surgery and perfusion from several polar arteries reducing visibility during resection, these challenging resections led to a greater resection margin than initially expected (Fig. 3). Despite omitting these two patients from the analysis, the LPN group still showed a greater SRF loss than the RFA group (adjusted difference = 2.1 percentage points, 95% CI = 0.58–3.57, *P* = 0.007).

There was no difference between the two groups in pre-treatment creatinine, but a slightly lower pre-treatment eGFR in the RFA group was observed. Kidney function was affected after treatment and was measured as an increase in creatinine and decrease in eGFR (Table 2). However, there was no difference between groups regarding the change in these values (Table 2).

## Discussion

Both RFA and LPN showed a high preservation of renal function when treating SRMs. However, RFA treatment was associated with a more favorable preservation of renal function of the treated kidney, measured as a greater reduction in SRF after LPN (Fig. 2 and Table 2).

Preservation of renal volume is one of the most influential factors on renal functional outcomes after PN (5,20–22). Woldu et al. (3) report a greater preservation of renal volume and significantly smaller change in GFR after TA than after PN; however, the quality of functioning nephrons within the preserved renal volume may not be homogenous. Zhu et al. (23) report a smaller decrease of SRF in their kidneys treated with RFA-assisted laparoscopic tumor enucleation than with LPN. The reduction of SRF in both their groups (RFA –9.4%, LPN –17%) (23) was greater than in this study (RFA –3.5%, LPN –5.7%) and could be partially explained by differences in treatment techniques. Also, their evaluation of SRF by renogram has its inherent limitations (9); therefore, measurement of the SRF on CE-CT may be a more accurate assessment of renal function (9,10).

Several reasons could account for the lower SRF loss after RFA than after LPN. Increasing tumor size and centrality are associated with increased loss of parenchymal volume (24); however, our treatment groups did not differ in these parameters. Percutaneous RFA is always performed under non-ischemic conditions; therefore, the temporary vascular clamping and tension applied on the renal parenchyma during renorrhaphy could result in a degree of renal hypotrophy with a further reduction in SRF after LPN. The wedge-shaped incision to remove the tumor in LPN (25) could entail a greater loss of nephrons than removing the tumor with a sphere-shaped ablation zone adapted to tumor shape and size. The higher complication rate in the LPN group (as reported in a previous study (15)) could add to further renal insult. Nevertheless, there was still a greater reduction of SRF after LPN in the comparative analysis and this difference remained significant after adjusted analysis and exclusion of the two LPN outliers. Some observations in the RFA group had a minimal increase in SRF after treatment (Fig. 2), which could be a result of measurement error and therefore reduce the mean group SRF change with RFA treatment.

Renal compensatory mechanisms and a normal contralateral kidney may mask the effect nephron-sparing procedures have on renal function (5). To overcome this problem, several studies focus on patients with single kidneys treated with PN or TA (4,26,27). Conflicting results on the effects of GFR and creatinine values after treatment and inclusion of varying tumor sizes, group differences in pre-treatment characteristics, and TA/PN techniques limit their conclusions (4,26,27).

The small difference in pre-treatment eGFR most likely reflects the age difference between the groups. The patients in the RFA group were older (Table 1), but the age difference was unlikely to explain the results, as they were adjusted for age in the multivariable analysis. Good pre-treatment serum creatinine and eGFR in both groups could partially explain why these values were not considerably affected after treatment and why there was no difference in the change of these values between treatment groups (5). However, a previous study reports that percutaneous ablation does not adversely impact renal function, even in patients with pre-existing chronic kidney disease (28).

As renal function decreases over time, a limitation to the present study was that post-treatment creatinine values were collected at varying time intervals for both treatment groups. Four patients in the RFA group required several treatment sessions to achieve complete tumor treatment, thus affecting the median time interval at which creatinine values were collected. The RFA group had the first follow-up CT slightly earlier than the LPN group, although this difference was minimal (three months). These time differences in data collection, unavoidable in a retrospective study, could have affected the outcome.

This single center retrospective study of our initial experience with both treatment methods has several limitations. Even though adjustment for tumor and patient characteristics was performed, patient and surgeon preferences could explain the findings. The study was further limited by exclusion of patients who could not undergo CE-CT, which could mask the loss of renal function in both groups. Serum creatinine was not performed at a single laboratory, which could have introduced errors into comparisons of creatinine and eGFR. The creatinine and GFR analyses were limited to a single post-treatment value, without evaluating long-term renal function. Other co-morbidities (e.g. diabetes, hypertension), the differing amount of contrast medium used in each treatment group, differences in method of anesthesia, or medications that might affect renal function were not assessed. Possible survival benefits correlated to changes in SRF were not evaluated, although this was not the primary goal. The SRF is not an absolute measurement of renal function; therefore, translation of the results to the individual patients’ renal function was not possible. CE-CT-based SRF measurement is a validated method for renal function assessment (9,12,14); however, this method could need further evaluation in patients treated for renal tumors. Nevertheless, RFA is associated with a more favorable preservation of renal function than LPN when measuring SRF. As SRMs are often found in older patients with pre-existing chronic kidney disease, preservation of renal function is vital in this group in order to minimize the development of end-stage kidney disease (29).

In conclusion, both RFA and LPN are good preservers of renal function when treating SRMs. In this series, RFA was associated with a more favorable preservation of renal function than LPN when assessing the effect of treatment on the affected kidney’s SRF. There was no difference in the change of creatinine and eGFR after treatment, which could be explained by the kidney’s compensatory mechanisms.

## Supplemental Material

sj-pdf-1-acr-10.1177_0284185120956281 - Supplemental material for Split renal function after treatment of small renal masses: comparison between radiofrequency ablation and laparoscopic partial nephrectomy Click here for additional data file. Supplemental material, sj-pdf-1-acr-10.1177_0284185120956281 for Split renal function after treatment of small renal masses: comparison between radiofrequency ablation and laparoscopic partial nephrectomy by Vanessa Acosta Ruiz, Sarah Båtelsson, Elina Onkamo, Lisa Wernroth, Thomas Nilsson, Maria Lönnemark, Pär Dahlman and Anders Magnusson in Acta Radiologica
