# Supplementary material for: Split renal function after treatment of small renal masses: comparison between radiofrequency ablation and laparoscopic partial nephrectomy
Source: Acta Radiol. 2020 Sep 10;62(9):1248–56. doi: 10.1177/0284185120956281 (PMC8392853; doi:10.1177/0284185120956281)
Supplement: sj-pdf-1-acr-10.1177_0284185120956281 - Supplemental material for Split renal function after treatment of small renal masses: comparison between radiofrequency ablation and laparoscopic partial nephrectomy [file sj-pdf-1-acr-10.1177_0284185120956281.pdf]

Supplementary table

Table 1. Number of excluded patients per treatment group, arranged according to frequency of patients not meeting the inclusion criteria; and frequency of patients following exclusion criteria.

Inclusion criteria	Number of excluded patients per treatment groups not following inclusion criteria	
	RFA Patients (n)	LPN Patients (n)
Single tumor	18	6
T1a tumors (≤4 cm)	5	13
Non-hereditary tumors	4	0
Renal tumor originating from the renal parenchyma	0	1
Tumor treatment with a curative intent	8	0
Patient age ≤75 years	8	0
Pre-treatment CE-CT images within a year prior and after treatment	14	10
CT images met the technical demands for image processing	12	7
Patients needed to have two kidneys	7	1
RFA performed under CT guidance with Cool-Tip RF ablation system	8	-
PN performed under laparoscopy	-	20
LPN converted to total nephrectomy	-	1
Follow-up showing 100% success rate during follow-up time	5	0
Exclusion criteria		
Tumors treated with multiple treatment methods	17	2
Total excluded	106	61
